# Supplementary figures and images for: Constructing a comprehensive gene co-expression based interactome in Bos taurus
Source: PeerJ. 2017 Dec 4;5:e4107. doi: 10.7717/peerj.4107 (PMC5719962; doi:10.7717/peerj.4107)

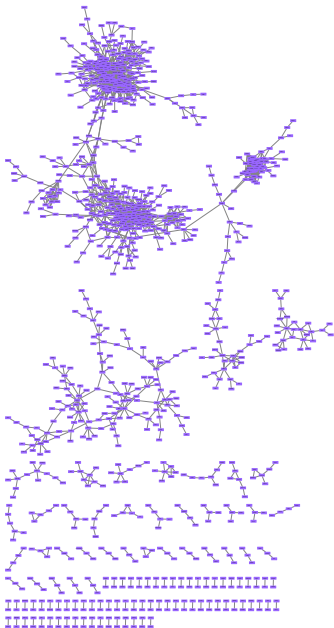

Supplement: Figure S1 [file peerj-05-4107-s001.pdf]
